# Kidney Function Decline in Sickle Cell Disease: Associations with Renin-Angiotensin System Inhibitors

**DOI:** 10.34067/KID.0000001116

**Published:** 2026-01-21

**Authors:** Kabir O. Olaniran, Alecia C. Nero, Orson W. Moe, Robert D. Toto, S. Susan Hedayati

**Affiliations:** 1 Department of Internal Medicine, University of Texas Southwestern Medical Center, Dallas, Texas; 2 Department of Physiology, University of Texas Southwestern Medical Center, Dallas, Texas; 3 Charles and Jane Pak Center for Mineral Metabolism and Clinical Research, University of Texas Southwestern Medical Center, Dallas, Texas; 4 Department of Population and Data Sciences, Peter O’Donnell Jr. School of Public Health, University of Texas Southwestern Medical Center, Dallas, Texas; 5 Department of Medicine, Stony Brook University, Stony Brook, New York

**Keywords:** renal function decline, renin-angiotensin system

## Abstract

**Key Points:**

- Renin-angiotensin system inhibitor use was not associated with slowed GFR decline in sickle cell disease.
- This null association did not change after excluding low-dose renin-angiotensin system inhibitors or including an interaction term with sickle cell disease therapies.
- Our findings highlight the limitations of real-world data and underscore the urgent need for prospective trials and novel therapeutics.

**Background:**

Sickle cell disease (SCD) is associated with accelerated kidney function decline, with no proven effective therapies. We examined the associations between treatment with renin-angiotensin system inhibitors (RASi) and eGFR decline in SCD.

**Methods:**

This two-center observational study used electronic health record data of adult, Black patients with SCD (by hemoglobin electrophoresis), and ≥1-year follow-up between 2010 and 2024. We compared incident RASi users (exposure) to no treatment (reference). We created 1:1 propensity score-matched cohorts, balancing on demographics, vital signs, comorbidities, medications, and laboratory values. The primary end point was the difference in the mean change in eGFR per year, analyzing only chronic slopes (≥90 days postindex date) using linear mixed models on the matched cohorts. Sensitivity analyses were performed excluding patients with missing albuminuria and excluding low-dose RASi. Effect modification by SCD-modifying therapies was also examined.

**Results:**

Matched cohorts identified were primary analysis (358 patients), excluding missing albuminuria data (262 patients), and excluding low-dose RASi (270 patients). All cohorts achieved optimal standardized mean differences <0.2. After multivariable adjustment, there was no significant difference in eGFR decline between RASi and the reference in the primary cohort (−0.15 ml/min per year; 95% confidence interval [CI], −1.67 to +1.36; *P* = 0.84), the sensitivity analysis cohort excluding missing albuminuria data (+0.89 ml/min per year; 95% CI, −0.86 to +2.63; *P* = 0.32), and the sensitivity analysis cohort excluding low-dose RASi (+0.78 ml/min per year; 95% CI, −1.12 to +2.67; *P* = 0.42). All *P* values for interaction terms between RASi and SCD-modifying therapies in all models were >0.05.

**Conclusions:**

In this large, real-world cohort of patients with SCD, RASi use was not associated with slowed eGFR decline. These findings underscore the limitations of observational data and highlight the urgent need for prospective trials to identify effective GFR-preserving therapies for this high-risk population.

## Visual Abstract

**Figure:**
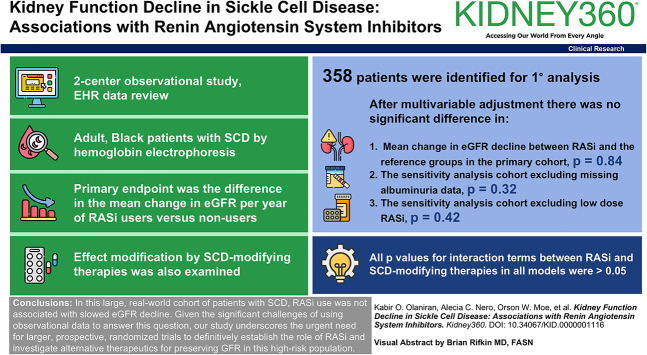

## Introduction

Sickle cell disease (SCD) is the most common inherited hematologic disorder in the world.^1^ Despite advances in SCD care, SCD is still associated with severe morbidity and early mortality, in a large part due to CKD.^1–4^ Remarkably, between 20% and 40% of all adults with SCD have CKD and nearly 20% of all SCD deaths can be attributed to CKD.^2,5^ Despite these dire findings, there are few studies on effective treatments for CKD in SCD.

Several studies have established the association between SCD and an increased risk for proteinuria and accelerated decline in eGFR.^6–9^ However, prospective studies involving CKD treatment in SCD have focused on the primary end point of proteinuria reduction with the use of renin-angiotensin system inhibitors (RASi) and hydroxyurea.^10–15^ These prospective studies were limited by small sample sizes and suboptimal follow-up for the evaluation of GFR decline. As a result, although RASi use is recommended for proteinuria reduction in SCD,^3,5^ the evidence for their benefit on accelerated eGFR decline in SCD is limited.^7,9^

The primary objective of this retrospective observational study was to compare the mean rate of change in eGFR among patients with SCD treated with RASi as compared with those not treated with RASi (no treatment). Secondary objectives included exploring the association between low-dose RASi use and GFR decline in SCD and interactions between RASi and SCD-modifying therapies.

## Methods

### Study Population

This study used electronic health record (EHR) data from the University of Texas Southwestern Medical Center (UTSW) and Parkland Health and Hospital Systems (PHHS) collected between January 1, 2010, and June 30, 2024. UTSW and PHHS EHR data are captured from inpatient and outpatient visits at the member hospitals and clinics of UTSW and PHHS. Both hospital systems are in Dallas-Fort Worth, Texas. Combined, UTSW and PHHS care for nearly six million patients annually. PHHS also serves as a safety net health system for indigent patients in the Dallas County area of Texas.

The Institutional Review Boards (IRB) at UTSW and PHHS, Dallas, Texas, reviewed and approved this study (IRB number STU-2021-0097). As part of this approval, the IRB granted a waiver of informed consent, determining that the research met the criteria for minimal-risk, retrospective analysis of data from the EHR.

### Inclusion and Exclusion Criteria

We applied the following inclusion criteria to extract data from the UTSW and PHHS EHRs: (*1*) SCD diagnosis codes (see Supplemental Appendix), (*2*) self-identified Black race, and (*3*) age ≥18 years at or after January 1, 2010. The following exclusion criteria were simultaneously applied: (*1*) <2 serum creatinine values available; (*2*) <1 year between the first and last serum creatinine values if ≥2 were available; (*3*) an eGFR <15 ml/min at first available serum creatinine; (*4*) RASi initiation before January 1, 2010; and (*5*) concurrent sickle cell trait diagnosis codes (see Supplemental Appendix).

All hemoglobin electrophoresis results were reviewed by one author (K.O. Olaniran) to ensure all participants have SCD.^16^ The reviewed hemoglobin electrophoresis tests were ordered as part of routine clinical care.

### Exposures

The index date for the exposure group was defined as the date of the first RASi prescription. We defined the primary exposure group as patients with a prescription for a RASi (see Supplemental Appendix) which was refilled at least once after 30 days.

The no treatment group (reference) was defined as patients who never received a prescription for any RASi during the study period. For the reference group, baseline was defined as the date of the first serum creatinine at or after January 1, 2010.

Low-dose RASi categories were defined based on the literature (equivalent of lisinopril ≤5 mg daily or losartan 12.5 mg daily).^17,18^

### Primary Outcome

The prespecified primary study outcome was the difference in the mean annual rate of change in eGFR. Only chronic slopes were analyzed by excluding all eGFR values between the index date and 90 days after the index date.^19^ The eGFR was calculated using the 2021 CKD-Epidemiology Collaboration (CKD-EPI) creatinine equation which did not adjust for race.^20^

### Covariates

Baseline covariates were assessed in the 3-month period before the index date. Demographics, vital signs, and smoking status were identified by manual chart review. Comorbidities (diabetes mellitus [DM], cardiovascular disease [CVD—stroke or coronary artery disease], and cardiopulmonary disease [CPD—heart failure or pulmonary hypertension]) were obtained using International Classification of Disease (ICD), Tenth edition, diagnosis codes and their ICD, Ninth edition equivalents (see Supplemental Appendix) obtained from encounter data and discharge data. Laboratory values at baseline and use of medications (sodium-glucose cotransporter 2 inhibitors [SGLT2i] or SCD-modifying therapies defined as any one or more of hydroxyurea, voxelotor, crizanlizumab, or apheresis/exchange transfusions/simple transfusions) at any time during follow-up were obtained by chart review. When only urine protein-to-creatinine ratios were available, they were converted to urine albumin-to-creatinine ratios (UACR) using previously published formulae.^21^

### Propensity Score Matching

**Figure 1:**
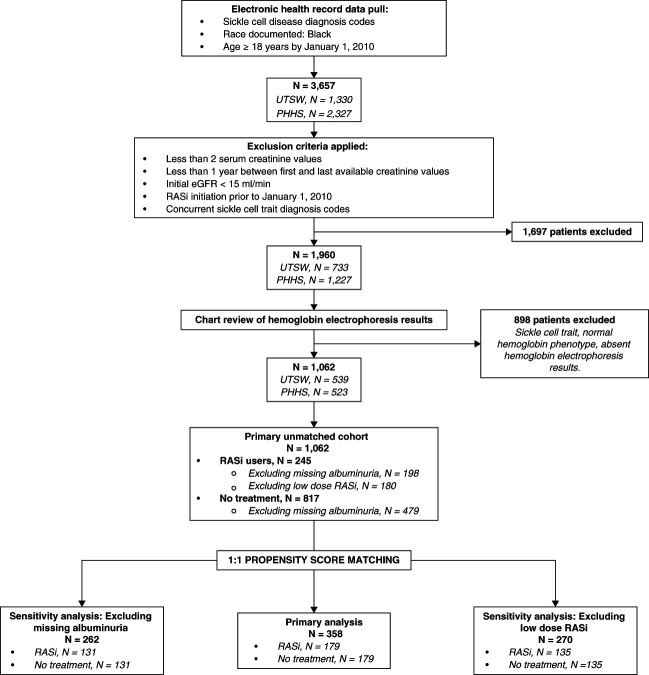
**Flow chart of inclusion in this study.** PHHS, Parkland Health and Hospital Systems; RASi, renin-angiotensin system inhibitors; UTSW, University of Texas Southwestern Medical Center.

We performed propensity score matching to address confounding by indication.^22^ Three cohorts were created (Figure 1): a primary analysis cohort, a sensitivity analysis cohort excluding patients with missing albuminuria data and a sensitivity analysis cohort excluding patients with low-dose RASi.^17,18^ The propensity score was calculated using a logistic regression model predicting incident RASi use. We matched each patient in the exposure group 1:1 without replacement to a patient in the no treatment group based on the propensity score (caliper width of 0.2 standard deviations of the logit of the estimated propensity score). The following covariates, selected *a priori*, were included in the multivariable logistic regression model: age, sex, sickle cell genotype, clinical site, systolic BP, diastolic BP, body mass index, DM, CVD, CPD, smoking status, baseline eGFR, albuminuria, baseline plasma hemoglobin concentration, and SCD-modifying therapies. SGLT2i were not included in the matching models due to sparse data and variable start dates long after the baseline. Baseline hemoglobin was preferred as an important clinical trial end point in SCD.^23^ Albuminuria was matched using baseline UACR categories (missing, <30, 30–299, or ≥300 mg/g) in the primary analysis cohort and low-dose RASi exclusion cohort. For the sensitivity analysis cohort excluding missing albuminuria data, we used log-transformed UACR for matching.

Matching was performed using *R*, version 4.4.2 (*R* Core Team, 2024).

### Statistical Analysis

Baseline characteristics were summarized using means with SDs (parametric continuous variables), median values with interquartile ranges (nonparametric continuous variables), and counts with percentages (categorical variables). We compared baseline propensity score matched characteristics using standardized mean differences (SMDs). Variables with an SMD <0.2 were considered to be well-matched.^24^

The primary outcome was modeled using linear mixed-effects models with random intercepts and random slopes for time at the subject level, thereby inducing the within-subject covariance structure through the random effects. To isolate the chronic medication effect from any acute hemodynamic drop, we restricted all models to eGFR values measured ≥90 days after the index date for both groups.

The primary effect was estimated from the interaction term between follow-up time and RASi use, which defines the difference in chronic eGFR slopes.^19^ To create a doubly robust estimate, all models were further adjusted for the baseline covariates used in the matching (age, sex, sickle cell genotype, clinical site, systolic BP, diastolic BP, body mass index, DM, CVD, CPD, smoking, SGLT2i use, SCD-modifying therapy, baseline eGFR, albuminuria, and baseline hemoglobin). Effect modification by SCD-modifying therapies was tested using a three-way interaction term (RASi, SCD-therapy, time).

Censorship occurred at death, dialysis initiation, or the last available serum creatinine date before June 30, 2024. All analyses were performed using the propensity-matched cohorts. The estimated difference in eGFR slopes, and their 95% confidence intervals (CI) were reported.

All analyses were conducted using STATA 18.5 (StataCorp., College Station, TX) and *R*, version 4.4.2 (*R* Core Team, 2024).

## Results

Selection of patients for the final unmatched cohort is presented in Figure 1. We identified 1062 patients (245 RASi and 817 no treatment).

### Primary Analysis Cohort: Baseline Characteristics and Annual Change in eGFR

Baseline characteristics of the RASi-matched cohorts are presented in Table 1. Before matching (SMD 1.56), RASi users were older with higher BPs, more prevalent comorbidities, SGLT2i use, SCD-modifying therapy use, and more severe albuminuria.

**Table 1:** Baseline characteristics of primary analysis cohort

Characteristics	Final Unmatched Cohort			RASi Matched Cohort		
	No Treatment	RASi	SMD	No Treatment	RASi	SMD
No. of patients	817	245	1.56	179	179	0.10
**Demographics**						
Mean age±SD, yr	28±10	40±13	1.06	37±12	37±12	0.01
Female, *N* (%)	471 (58%)	129 (53%)	−0.05	101 (56%)	95 (53%)	−0.03
SS Genotype, *N* (%)	573 (70%)	165 (67%)	−0.03	124 (69%)	126 (70%)	0.01
Safety net hospital clinic, *N* (%)	397 (49%)	126 (51%)	−0.03	84 (47%)	90 (50%)	0.03
Median follow-up (IQR), yr^a^	6.2 (3.6–9.9)	4.3 (1.9–9.7)	N/A	6.9 (3.9–11.3)	3.8 (1.7–8.8)	N/A
Mean systolic BP±SD, mm Hg	121±16	132±21	0.56	127±17	129±19	0.14
Mean DBP±SD, mm Hg	71±12	78±12	0.56	76±11	77±12	0.06
Mean BMI±SD, kg/m^2^	24±6	28±7	0.48	27±7	27±6	−0.04
**Comorbidities**						
DM, *N* (%)	72 (9%)	50 (20%)	0.12	30 (17%)	24 (13%)	−0.03
Stroke or coronary artery disease, *N* (%)	96 (12%)	46 (19%)	0.07	32 (18%)	31 (17%)	−0.01
Heart failure or pulmonary hypertension, *N* (%)	281 (34%)	108 (44%)	0.10	73 (41%)	75 (42%)	0.01
Smoking, *N* (%)	57 (7%)	18 (7%)	0.00	16 (9%)	11 (6%)	−0.03
**Lab values**						
Mean eGFR±SD (ml/min per 1.73 m^2^)	122±22	104±27	−0.68	110±26	108±27	−0.10
eGFR categories, *N* (%)			N/A			N/A
≥9*0 ml/min*	734 (89%)	174 (71%)		138 (77%)	135 (75%)	
*60–89 ml/min*	68 (8%)	51 (21%)		32 (18%)	31 (17%)	
*30–59 ml/min*	12 (2%)	17 (7%)		6 (3%)	12 (7%)	
*15–29 ml/min*	3 (1%)	3 (1%)		3 (2%)	1 (1%)	
Urine albumin-to-creatinine ratio, *N* (%)						
*≥300 mg/g*	34 (4%)	58 (24%)	0.20	26 (15%)	37 (21%)	0.06
*30–299 mg/g*	159 (20%)	81 (33%)	0.14	60 (34%)	53 (30%)	−0.04
*<30 mg/g*	286 (35%)	59 (24%)	−0.11	47 (26%)	47 (26%)	0.00
*Missing*	338 (41%)	47 (19%)	−0.22	46 (26%)	42 (24%)	−0.02
Mean plasma hemoglobin±SD, g/dl	9.5±1.9	9.6±2.0	0.09	9.5±2.0	9.6±2.0	0.03
**Other**						
Sickle cell disease-modifying therapies, *N* (%)	491 (60%)	166 (68%)	0.08	121 (67%)	121 (67%)	0.00
SGLT2i during follow up, *N* (%)^a^	2 (0.2%)	29 (12%)	N/A	1 (1%)	16 (9%)	N/A

Sickle cell disease-modifying therapies: any one or more of hydroxyurea, voxelotor, crizanlizumab, or apheresis/exchange transfusions/simple transfusions.

BMI, body mass index; DBP, diastolic BP; DM, diabetes mellitus; IQR, interquartile range; RASi, renin-angiotensin system inhibitors; SGLT2i, sodium-glucose cotransporter 2 inhibitors; SMD, standardized mean difference; SS, homozygous sickle cell anemia.

a Variable was not matched on, only adjusted for in final models.

After propensity score matching, 358 patients were identified for primary analysis (Table 1). Characteristics were well balanced between RASi and no treatment (SMD 0.10). The primary analysis cohort median follow-up time was 5.4 years (interquartile range, 2.8–10.1), with a mean age of 37±12 years, 55% female, and a mean baseline eGFR of 109±27 ml/min. Forty-nine percent of patients had a UACR ≥30 mg/g, and the mean hemoglobin was 9.5±2.0 g/dl. Fifteen percent had DM, 18% had CVD, 41% had CPD, and 70% were hemoglobin homozygous sickle cell anemia or S-*β* thalassemia zero genotype. Supplemental Table 1 presents the frequency of SCD genotypes. Sixty-eight percent were on SCD-modifying therapies which sometimes overlapped (237 hydroxyurea, 31 voxelotor, two crizanlizumab, and 18 exchange transfusions/apheresis/simple transfusions).

The matched primary analysis cohort of 358 patients had a median of 25 (interquartile range, 11–74) eGFR values. The adjusted mean annual change in eGFR in the matched primary analysis cohort was −2.98 (95% CI, −4.05 to −1.91) ml/min per year.

**Figure 2:**
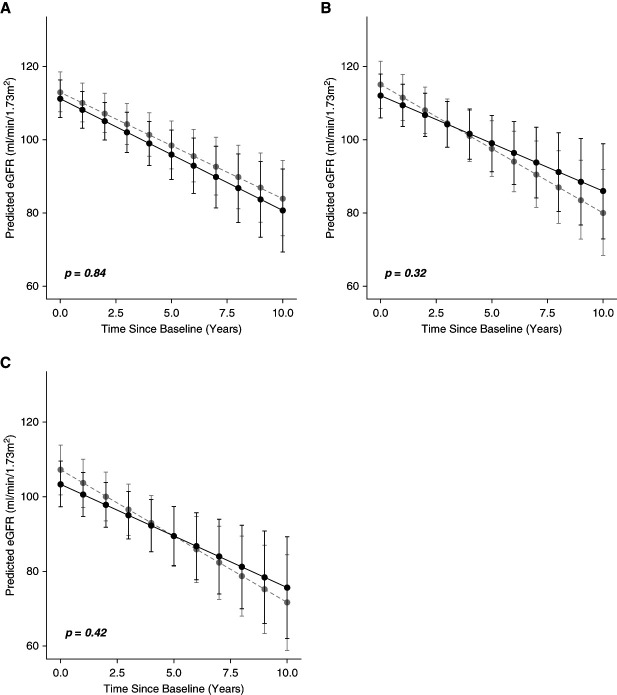
**Renin-angiotensin system Inhibitor use was not associated with slower eGFR decline.** Predicted slopes of the adjusted mean change in eGFR over study period in (A) the primary analysis cohort, (B) the sensitivity analysis cohort excluding missing albuminuria data, and (C) the sensitivity analysis cohort excluding low-dose RASi.

RASi use was not associated with significantly faster or slower eGFR decline (−0.15 ml/min per year; 95% CI, −1.67 to +1.36) compared with no treatment (Figure 2A and Table 2). No interaction was observed between RASi use and SCD-modifying therapies in relation to eGFR decline in this cohort.

**Table 2:** Difference in mean eGFR change per year in renin-angiotensin system inhibitors compared with no treatment

	Median Number of eGFR Values (IQR)	Unadjusted Difference in the Mean Annual Change in eGFR, *β* (95% CI)	Adjusted Difference in the Mean Annual Change in eGFR, *β* (95% CI)	Adjusted Mean Annual Change in eGFR, *β* (95% CI)
**Primary matched cohort**				
No treatment	31 (14–87)	0	0	−2.90 (−3.90 to −1.90)
RASi	18 (9–61)	−0.25 (−1.79 to +1.29) *P* = 0.75	−0.15 (−1.67 to +1.36)^a^ *P* = 0.84	−3.05 (−4.19 to −1.91)
RASi+SCD-modifying therapy	N/A	−0.21 (−3.59 to +3.17) *P* = 0.90	−0.22 (−3.54 to+3.11)^a^ *P* = 0.90	N/A
**Sensitivity analysis: excluding missing albuminuria data**				
No treatment	45 (17–96)	0	0	−3.49 (−4.63 to −2.35)
RASi	22 (10–68)	+0.89 (−0.88 to +2.65) *P* = 0.34	+0.89 (−0.86 to +2.63)^b^* P* = 0.32	−2.61 (−3.92 to −1.29)
RASi+SCD-modifying therapy	N/A	+0.38 (−3.68 to +4.44) *P* = 0.86	+0.37 (−3.62 to +4.36)^b^* P* = 0.86	N/A
**Sensitivity analysis: excluding low-dose RASi**				
No treatment	31 (12–81)	0	0	−3.55 (−4.82 to −2.28)
RASi	22 (10–66)	+0.75 (−1.17 to +2.67) *P* = 0.45	+0.78 (−1.12 to +2.67)^a^ *P* = 0.42	−2.78 (−4.18 to −1.37)
RASi+SCD-modifying therapy	N/A	+0.95 (−3.20 to +5.09) *P* = 0.65	+1.03 (−3.05 to +5.12)^a^ *P* = 0.62	N/A

CI, confidence interval; IQR, interquartile range; RASi, renin-angiotensin system inhibitors; SCD, sickle cell disease.

a Adjusted for age, sex, sickle cell genotype, safety net hospital clinics, systolic BP, diastolic BP, body mass index, diabetes mellitus, stroke or coronary artery disease, heart failure or pulmonary hypertension, smoking, sodium-glucose cotransporter 2 inhibitors use, sickle cell disease-modifying therapy, baseline eGFR value, baseline urine albumin-to-creatinine ratio category, and baseline hemoglobin.

b Adjusted for age, sex, sickle cell genotype, safety net hospital clinics, systolic BP, diastolic BP, body mass index, diabetes mellitus, stroke or coronary artery disease, heart failure or pulmonary hypertension, smoking, sodium-glucose cotransporter 2 inhibitors use, sickle cell disease modifying therapy, baseline eGFR value, baseline log-transformed urine albumin-to-creatinine ratio, and baseline hemoglobin.

### Sensitivity Analysis Cohort: Exclusion of Patients with Missing Albuminuria Data

After excluding patients with missing albuminuria data, baseline characteristics were well matched (262 patients, SMD 0.08), as presented in Supplemental Table 2. The use of RASi was again, not significantly associated with either faster or slower eGFR decline in SCD compared with the reference (Figure 2B and Table 2). As in the primary cohort, there was no evidence that the effect of RASi on eGFR decline was modified by SCD-modifying therapies (Table 2).

### Sensitivity Analysis: Excluding Patients on Low-Dose RASi

The highest dose of RASi prescribed is presented in Supplemental Table 3. Twenty-eight percent of RASi users were on an equivalent dose of ≤lisinopril 5 mg or losartan 12.5 mg daily (low dose).^17,18^ Baseline characteristics for RASi excluding low dose are presented in Supplemental Table 4. Sensitivity analysis excluding low-dose RASi (Figure 2C and Table 2) showed that there was no significant difference in eGFR decline between RASi use and no treatment as well as in RASi use with SCD-modifying therapies.

## Discussion

To the best of our knowledge, this is the largest longitudinal study to date to investigate the association of RASi use with changes in eGFR over time in patients with SCD verified by hemoglobin electrophoresis. In our primary analysis, we found no significant association between RASi use and the rate of eGFR decline. This finding was robust across key sensitivity analyses, including those restricted to patients with complete baseline albuminuria data and those excluding patients on low-dose RASi. However, this primary finding must be interpreted with significant caution. Despite rigorous propensity score matching, we observed residual confounding by indication, most notably a higher baseline burden of albuminuria in the RASi group. As this baseline imbalance indicates that the RASi group was at a higher intrinsic risk for progression, it fundamentally limits the interpretation of our null finding.

RASi are recommended for the slowing of eGFR decline in the at risk/CKD population,^25,26^ but evidence for this in SCD is very limited. Kidney disease in SCD is pathophysiologically distinct from several other forms of CKD.^3,4^ Two small, prospective studies in SCD with losartan showed no benefit on slowing GFR decline over 6 months and 12 months, respectively.^10,14^ Only one retrospective study of 154 SCD patients (56% RASI) showed that RASi was associated with 1.9 ml/min slower eGFR decline over a median of 2.2 years in SCD.^27^ However, propensity score matching was not performed in this retrospective study, and variable start times for RASi (including preceding study onset time) were reported.^27^ This study also did not assess the effect of RASi doses or interactions with SCD-modifying therapies. By contrast, our study included a larger population followed over a median of nearly 5 years and used propensity scoring matching methodology to more rigorously analyze outcomes. Our findings therefore align with the limited prospective data. This suggests that the GFR-protective benefit of RASi observed in other high-risk CKD populations may not be directly generalizable to a broader SCD cohort where the primary pathophysiologic drivers include hemolysis-driven endothelial dysfunction, renal medullary and cortical recurrent ischemia-reperfusion injuries, cortical iron deposition, and hemolysis/PG-driven glomerular hyperfiltration.^3,4^

We must acknowledge the challenge of confounding by indication in this analysis. Despite matching, the RASi group had a higher baseline burden of albuminuria, a key driver of GFR decline. This context is critical for interpreting our findings. The fact that the higher-risk, RASi group experienced a rate of eGFR decline that was not significantly different from the lower-risk, matched-control group could be cautiously viewed as hypothesis-generating. It may suggest a potential, albeit unmeasured, blunting of their expected faster decline. However, our study was not designed to confirm this, and the finding remains inconclusive. Our results do not challenge the current American Society of Hematology recommendations, which are based on prospective evidence for albuminuria reduction. Our findings do, however, highlight the limitations of RASi in this population and underscore the need for alternative therapies for GFR decline in SCD to be investigated in future prospective studies.

The BP lowering effect of RASi is an important limitation. SCD is associated with significantly lower BPs compared with the general population (in part due to low peripheral vascular resistance).^28,29^ This represents a major clinical barrier, as prescribers in real-world settings must often limit RASi up-titration to avoid hypotension. This challenge, however, does not fully explain our findings. In the clinical trials examining albuminuria in SCD, higher doses (equivalent to losartan 50 mg or more) were targeted over 6–12 months but still did not observe changes in GFR decline.^10,14^ Our sensitivity analysis excluding low-dose RASi appears to support these prospective study findings.

This study's primary strength is that it is the largest longitudinal cohort study published to date investigating the association of RASi use with change in eGFR in patients with SCD, where the diagnosis of SCD was confirmed by hemoglobin electrophoresis. Our study design, which included only incident RASi users and used multiple eGFR data points per patient over a median follow-up of 4.7 years, allowed for robust longitudinal modeling. We applied rigorous propensity score matching to mitigate confounding by indication, a critical problem in SCD. Furthermore, the overall eGFR decline observed in our cohort is consistent with rates reported in other US SCD cohorts, supporting the generalizability of our findings.^7,9^ In the United States, SCD overwhelmingly affects individuals of African descent. Our study population, sourced from our health systems, reflects this established epidemiology.^30^

Our study has several important limitations. The primary limitation is the residual confounding by indication that persists despite matching. Although most covariates were well balanced (SMD <0.2), we observed a suboptimal balance in albuminuria, with the RASi group having a higher burden of this key risk factor. This imbalance suggests that the RASi group was at a higher intrinsic risk for progression and fundamentally limits the interpretation of our null finding. Second, we were unable to reliably evaluate longitudinal changes in albuminuria due to the sparse nature of follow-up UACR data. Third, we were unpowered to reliably analyze hard end points such as incident stage 5 CKD and mortality due to the modest number of events. Finally, owing to the retrospective nature of this study, we could not verify medication adherence, capture changes in RASi doses over time, ascertain APOL1 genotype status, account for all potential medication confounders (such as mineralocorticoid receptor agonists or glucagon-like receptor-1 agonist use), and we relied on ICD codes for comorbidities, which may be subject to misclassification.

In conclusion, our large, real-world study of patients with SCD found no significant association between RASi use and the rate of eGFR decline. This finding was robust across sensitivity analyses but must be interpreted cautiously given residual confounding by indication. Our results, which align with limited prospective trial data, suggest that the GFR-protective benefits of RASi seen in other high-risk CKD populations may not be directly generalizable to a broader SCD cohort, particularly one with mostly preserved kidney function. Given the significant challenges of using observational data to answer this question, our study underscores the urgent need for larger, prospective, randomized trials to definitively establish the role of RASi and investigate alternative therapeutics for preserving GFR in this high-risk population.

## Supplementary Material

## Data Availability

Original data generated for the study will be made available upon reasonable request to the corresponding author. Data Type: Aggregated Data; Observational Data. Reason for Restricted Access: Private patient health information obtained from EHRs.

## Declarative Statements

This study is exempt from the Institutional Review Board or Ethics Committee approval, and I have provided the reason for exemption. Minimal risk. The clinical and research activities being reported are consistent with the Principles of the Declaration of Istanbul as outlined in the Declaration of Istanbul on Organ Trafficking and Transplant Tourism.

## Supplemental Material

Supplemental Table 1. Distribution of SCD genotypes in the primary unmatched and primary analysis cohort.

Supplemental Table 2. Baseline characteristics of sensitivity analysis cohort excluding missing albuminuria data.

Supplemental Table 3. Distribution of dose categories of RASi.

Supplemental Table 4. Baseline characteristics of sensitivity analysis cohort excluding low-dose RASI users.
