# Supplementary material for: Kidney Function Decline in Sickle Cell Disease: Associations with Renin-Angiotensin System Inhibitors
Source: Kidney360. 2026 Jan 21;7(5):1056–64. doi: 10.34067/KID.0000001116 (PMC13229428; doi:10.34067/KID.0000001116)
Supplement: Supplementary file 1 [file kidney360-7-1056-s001.pdf]

ASN
American Society of Nephrology
JASN
Journal of the American Society of Nephrology
KIDNEY360
Accessing Our World From Every Angle
CJASN
Clinical Journal of the American Society of Nephrology

ASN Journal Disclosure Form

As per ASN journal policy, I have disclosed any financial relationships or commitments I have held in the past 36 months as included below. I have listed my Current Employer below to indicate there is a relationship requiring disclosure. If no relationship exists, my Current Employer is not listed.

S. Hedayati reports the following:
Employer: Stony Brook University, Renaissance School of Medicine; University of Texas Southwestern Medical Center; Consultancy: Medscape; and Other Interests or Relationships: ACP MKSAP Nephrology writing committee.

I understand that the information above will be published within the journal article, if accepted, and that failure to comply and/or to accurately and completely report the potential financial conflicts of interest could lead to the following: 1) Prior to publication, article rejection, or 2) Post-publication, sanctions ranging from, but not limited to, issuing a correction, reporting the inaccurate information to the authors' institution, banning authors from submitting work to ASN journals for varying lengths of time, and/or retraction of the published work.

Name: Susan Hedayati
Manuscript ID: K360-2025-000834R1
Manuscript Title: Kidney Function Decline in Sickle Cell Disease: Associations with Renin Angiotensin System Inhibitors
Date of Completion: October 28, 2025
Disclosure Updated Date: October 28, 2025

ASN American Society of Nephrology JASN Journal of the American Society of Nephrology KIDNEY360 Accessing Our World From Every Angle CJASN Clinical Journal of the American Society of Nephrology

ASN Journal Disclosure Form

As per ASN journal policy, I have disclosed any financial relationships or commitments I have held in the past 36 months as included below. I have listed my Current Employer below to indicate there is a relationship requiring disclosure. If no relationship exists, my Current Employer is not listed.

O. Moe reports the following:
Employer: University of Texas Southwestern Medical Center; Consultancy: Epok therapeutics; Research Funding: EPOK Therapeutics; Honoraria: Alnylam- Consultation fees; Advisory or Leadership Role: Editor, Seldin & Giebisch The Kidney ; Editor, Current Opinion of Nephrology and Hypertension;; and Other Interests or Relationships: American Society of Nephrology, National Kidney Foundation, American Physiology Society, International Society of Nephrology.

I understand that the information above will be published within the journal article, if accepted, and that failure to comply and/or to accurately and completely report the potential financial conflicts of interest could lead to the following: 1) Prior to publication, article rejection, or 2) Post-publication, sanctions ranging from, but not limited to, issuing a correction, reporting the inaccurate information to the authors' institution, banning authors from submitting work to ASN journals for varying lengths of time, and/or retraction of the published work.

Name: Orson W. Moe
Manuscript ID: K360-2025-000834R1
Manuscript Title: Kidney Function Decline in Sickle Cell Disease: Associations with Renin Angiotensin System Inhibitors
Date of Completion: December 16, 2025
Disclosure Updated Date: December 16, 2025

ASN American Society of Nephrology
JASN Journal of the American Society of Nephrology
KIDNEY360 Accessing Our World From Every Angle
CJASN Clinical Journal of the American Society of Nephrology

ASN Journal Disclosure Form

As per ASN journal policy, I have disclosed any financial relationships or commitments I have held in the past 36 months as included below. I have listed my Current Employer below to indicate there is a relationship requiring disclosure. If no relationship exists, my Current Employer is not listed.

A. Nero reports the following:
Employer: UT Southwestern Medical Center; Consultancy: Novo Nordisk; Fulcrum Therapeutics; Editas Medicine;; and Research Funding: Pfizer.

I understand that the information above will be published within the journal article, if accepted, and that failure to comply and/or to accurately and completely report the potential financial conflicts of interest could lead to the following: 1) Prior to publication, article rejection, or 2) Post-publication, sanctions ranging from, but not limited to, issuing a correction, reporting the inaccurate information to the authors' institution, banning authors from submitting work to ASN journals for varying lengths of time, and/or retraction of the published work.

Name: Alecia Nero
Manuscript ID: K360-2025-000834R1
Manuscript Title: Kidney Function Decline in Sickle Cell Disease: Associations with Renin Angiotensin System Inhibitors
Date of Completion: November 9, 2025
Disclosure Updated Date: November 9, 2025

ASN American Society of Nephrology | JASN Journal of the American Society of Nephrology | KIDNEY360 Accessing Our World From Every Angle | CJASN Clinical Journal of the American Society of Nephrology

ASN Journal Disclosure Form

As per ASN journal policy, I have disclosed any financial relationships or commitments I have held in the past 36 months as included below. I have listed my Current Employer below to indicate there is a relationship requiring disclosure. If no relationship exists, my Current Employer is not listed.

K. Olaniran has nothing to disclose.

I understand that the information above will be published within the journal article, if accepted, and that failure to comply and/or to accurately and completely report the potential financial conflicts of interest could lead to the following: 1) Prior to publication, article rejection, or 2) Post-publication, sanctions ranging from, but not limited to, issuing a correction, reporting the inaccurate information to the authors' institution, banning authors from submitting work to ASN journals for varying lengths of time, and/or retraction of the published work.

Name: Kabir O. Olaniran
Manuscript ID: K360-2025-000834R1
Manuscript Title: Kidney Function Decline in Sickle Cell Disease: Associations with Renin Angiotensin System Inhibitors
Date of Completion: December 16, 2025
Disclosure Updated Date: December 16, 2025

ASN
American Society of Nephrology
JASN
Journal of the American Society of Nephrology
KIDNEY360
Accessing Our World From Every Angle
CJASN
Clinical Journal of the American Society of Nephrology

ASN Journal Disclosure Form

As per ASN journal policy, I have disclosed any financial relationships or commitments I have held in the past 36 months as included below. I have listed my Current Employer below to indicate there is a relationship requiring disclosure. If no relationship exists, my Current Employer is not listed.

R. Toto reports the following:
Employer: University of Texas Southwestern Medical Center; Consultancy: Astra-Zeneca, Boehringer-Ingelheim, Alnylm Pharma, EpoK Pharma; Research Funding: NIH; Vertex pharma; Honoraria: Astra-Zeneca, Alnylam Pharma, Novo Nordisk; and Advisory or Leadership Role: Astra-Zeneca, Boehringer-Ingelheim,Novo Nordisk, EpoK.

I understand that the information above will be published within the journal article, if accepted, and that failure to comply and/or to accurately and completely report the potential financial conflicts of interest could lead to the following: 1) Prior to publication, article rejection, or 2) Post-publication, sanctions ranging from, but not limited to, issuing a correction, reporting the inaccurate information to the authors' institution, banning authors from submitting work to ASN journals for varying lengths of time, and/or retraction of the published work.

Name: Robert D. Toto
Manuscript ID: K360-2025-000834R1
Manuscript Title: Kidney Function Decline in Sickle Cell Disease: Associations with Renin Angiotensin System Inhibitors
Date of Completion: January 7, 2026
Disclosure Updated Date: January 7, 2026
