# Supplementary material for: Kidney Function Decline in Sickle Cell Disease: Associations with Renin-Angiotensin System Inhibitors
Source: Kidney360. 2026 Jan 21;7(5):1056–64. doi: 10.34067/KID.0000001116 (PMC13229428; doi:10.34067/KID.0000001116)
Supplement: Supplementary file 2 [file kidney360-7-1056-s002.pdf]

SUPPLEMENTAL MATERIAL

Supplemental Table 1. Distribution of sickle cell disease genotypes in the primary unmatched and primary analysis cohort.
Supplemental Table 2. Baseline characteristics of sensitivity analysis cohort excluding missing albuminuria data.
Supplemental Table 3. Distribution of dose categories of renin angiotensin system inhibitors (RASi).
Supplemental Table 4. Baseline characteristics of sensitivity analysis cohort excluding low dose renin angiotensin system inhibitors users.

Supplemental table 1. Distribution of sickle cell disease genotypes in the primary unmatched and primary analysis cohort.

Genotype	Primary unmatched cohort<br>N = 1,062	Primary analysis cohort<br>N = 358
Hb SS	732	250
Hb SC	259	84
Hb S beta thalassemia zero	6	2
Hb S beta thalassemia plus	59	19
Hb SS with hereditary persistent fetal hemoglobin	2	2
Hb SD	1	0
Hb SE	1	1
Hb SO-Arab	2	0

Supplemental Table 2. Baseline characteristics of sensitivity analysis cohort excluding missing albuminuria data.

Characteristics	UNMATCHED COHORT Excluding missing albuminuria data			SENSITIVITY ANALYSIS COHORT Excluding missing albuminuria data		
	**No treatment**	**RASi**	**SMD**	**No treatment**	**RASi**	**SMD**
**No. of patients**	**479**	**198**	**1.54**	**131**	**131**	**0.08**
Demographics						
Mean age ± SD, years	27 ± 10	40 ± 12	1.10	35 ± 12	36 ± 11	0.07
Female, N (%)	282 (59%)	105 (53%)	-0.06	72 (55%)	71 (54%)	-0.01
SS genotype, N (%)	346 (72%)	137 (69%)	-0.03	97 (74%)	94 (72%)	-0.02
Safety net hospital clinic, N (%)	221 (46%)	105 (53%)	-0.07	60 (46%)	60 (46%)	0.00
Median follow-up (IQR), years*	7.4 (4.7 to 11.7)	4.3 (1.9 to 9.5)	N/A	8.7 (5.0 to 12.3)	4.0 (1.8 to 8.8)	N/A
Mean systolic blood pressure ± SD, mmHg	120 ± 15	132 ± 21	0.64	125 ± 17	126 ± 16	0.04
Mean diastolic blood pressure ± SD, mmHg	70 ± 11	78 ± 12	0.65	75 ± 11	75 ± 11	0.02
Mean body mass index ± SD, kg/m^2	24 ± 5	27 ± 7	0.57	26 ± 6	26 ± 6	0.08
Comorbidities						
Diabetes mellitus, N (%)	61 (13%)	41 (21%)	0.08	17 (13%)	24 (18%)	0.05
Stroke or coronary artery disease, N (%)	68 (14%)	35 (18%)	0.04	24 (18%)	24 (18%)	0.00
Heart failure or pulmonary hypertension, N (%)	159 (33%)	82 (41%)	0.08	49 (37%)	51 (39%)	0.02
Smoking, N (%)	32 (7%)	11 (6%)	-0.01	7 (5%)	8 (6%)	0.01
Lab values						
Mean eGFR±SD (mL/min/BSA)	123 ± 21	104 ± 27	-0.81	112 ± 28	111 ± 25	-0.05
eGFR categories, N (%)			N/A			N/A
≥90 ml/min	437 (91%)	144 (73%)		104 (79%)	109 (83%)	
60–89 ml/min	35 (7%)	39 (20%)		20 (15%)	14 (11%)	
30–59 ml/min	4 (1%)	13 (6%)		4 (3%)	7 (5%)	
15–29 ml/min	3 (1%)	2 (1%)		3 (2%)	1 (1%)	
Urine albumin to creatinine ratio (IQR), mg/g [a]	29 (10 to 81)	104 (17 to 358)	0.56	54 (16 to 144)	69 (16 to 289)	-0.02
Urine albumin to creatinine ratio, N (%)			N/A			N/A
≥300 *mg/g*	34 (7%)	58 (29%)		24 (18%)	33 (25%)	
30 to 299 mg/g	159 (33%)	81 (41%)		53 (41%)	55 (42%)	
<30 mg/g	286 (60%)	59 (30%)		54 (41%)	43 (33%)	
Mean plasma hemoglobin ± SD, g/dL	9.4 ± 2.0	9.6 ± 2.1	0.08	9.3 ± 2.0	9.5 ± 2.0	0.11
Other						
Sickle cell disease modifying medications, N (%)	300 (63%)	140 (71%)	0.08	98 (75%)	92 (70%)	-0.05
SGLT2i, N (%) [b]	2 (0.4%)	28 (14%)	N/A	2 (2%)	17 (13%)	N/A

[a] Exponentiated after matching performed on log-transformed values.
[b] Variable was not matched on, only adjusted for in final models.
RASi: renin angiotensin system inhibitors. SMD: standardized mean difference. eGFR: estimated glomerular filtration rate. SGLT2i: sodium-glucose cotransporter-2 inhibitors. Sickle cell disease modifying therapies: any one or more of hydroxyurea, voxelotor, crizanlizumab, or apheresis/exchange transfusions/simple transfusions.

Supplemental table 3. Distribution of dose categories of renin angiotensin system inhibitors (RASi).

Cohort	Low dose RASi N (%)	Moderate dose RASi N (%)	Maximum dose RASi N (%)
**Primary analysis cohort** *N = 179*	50 (28%)	106 (59%)	23 (13%)
**Sensitivity analysis cohort excluding albuminuria** *N = 131*	38 (29%)	76 (58%)	17 (13%)
**Sensitivity analysis cohort excluding low dose RASi** *N = 135*	N/A	114 (84%)	21 (16%)

Low dose RASi: Lisinopril (or ACEi) equivalent) ≤ 5 mg daily or Losartan (or ARB equivalent) ≤ 12.5 mg daily

Moderate dose RASi: Lisinopril (or ACEi equivalent) 10 – 30 mg daily or Losartan (or ARB equivalent) 25 – 75 mg daily.

Maximal dose RASi: Lisinopril (or ACEi equivalent) ≥ 40 mg daily or Losartan (or ARB equivalent) ≥ 100 mg daily.

RASi - renin angiotensin system inhibitors, ARB - angiotensin receptor blockers, ACEi - angiotensin converting enzyme inhibitors.

Supplemental table 4. Baseline characteristics of sensitivity analysis cohort excluding low dose renin angiotensin system inhibitors users.

Characteristics	UNMATCHED COHORT Excluding low dose RASi			SENSITIVITY ANALYSIS COHORT Excluding low dose RASi		
	No treatment	RASi	SMD	No treatment	RASi	SMD
**No. of patients**	**817**	**180**	**1.66**	**135**	**135**	**0.07**
Demographics						
Mean age ± SD, years	28 ± 10	42 ± 12	1.20	39 ± 13	39 ± 12	-0.001
Female, N (%)	471 (58%)	98 (54%)	-0.03	69 (51%)	76 (56%)	0.05
SS genotype, N (%)	573 (70%)	115 (64%)	-0.06	90 (67%)	90 (67%)	0.00
Safety net hospital clinic, N (%)	397 (49%)	84 (47%)	0.02	75 (56%)	72 (53%)	-0.02
Median follow-up (IQR), years*	6.2 (3.6 to 9.9)	5.0 (1.9 to 10.8)	N/A	6.4 (3.6 to 11.5)	5.2 (1.9 to 12.0)	N/A
Mean systolic blood pressure ± SD, mmHg	121 ± 16	135 ± 20	0.76	131 ± 18	132 ± 20	0.06
Mean diastolic blood pressure ± SD, mmHg	71 ± 12	79 ± 12	0.69	78 ± 12	78 ± 13	0.02
Mean body mass index ± SD, kg/m^2	24 ± 6	28 ± 7	0.59	28 ± 7	28 ± 7	0.03
Comorbidities						
Diabetes mellitus, N (%)	72 (9%)	40 (22%)	0.13	21 (16%)	24 (18%)	0.02
Stroke or coronary artery disease, N (%)	96 (12%)	35 (19%)	0.08	22 (16%)	23 (17%)	0.01
Heart failure or pulmonary hypertension, N (%)	281 (34%)	77 (43%)	0.08	57 (42%)	59 (44%)	0.02
Smoking, N (%)	57 (7%)	15 (8%)	0.01	11 (8%)	11 (8%)	0.00
Lab values						
Mean eGFR±SD (mL/min/1.73m^2)	122 ± 22	100 ± 27	-0.84	106 ± 28	104 ± 26	-0.08
eGFR categories, N (%)			N/A			N/A
≥90 ml/min	734 (90%)	119 (66%)		98 (73%)	99 (73%)	
60–89 ml/min	68 (8%)	44 (24%)		28 (21%)	24 (18%)	
30–59 ml/min	12 (1%)	14 (8%)		6 (4%)	11 (8%)	
15–29 ml/min	3 (1%)	3 (2%)		3 (2%)	1 (1%)	
Urine albumin to creatinine ratio, N (%)						
≥300 *mg/g*	34 (4%)	42 (23%)	0.19	23 (17%)	26 (19%)	0.02
30–299 mg/g	159 (20%)	56 (31%)	0.12	38 (28%)	37 (27%)	-0.01
<30 mg/g	286 (35%)	47 (26%)	-0.09	42 (31%)	39 (29%)	-0.02
Missing	338 (41%)	35 (20%)	-0.22	32 (24%)	33 (24%)	0.01
Mean plasma hemoglobin ± SD, g/dL	9.5 ± 1.9	9.8 ± 2.1	0.19	9.7 ± 2.2	9.6 ± 2.1	-0.06
Other						
Sickle cell disease modifying medications, N (%)	491 (60%)	120 (67%)	0.07	92 (68%)	91 (67%)	-0.01
SGLT2i, N (%) [a]	2 (0.2%)	23 (13%)	N/A	1 (1%)	15 (11%)	N/A

[a] Variable was not matched on, only adjusted for in final models.

RASi: renin angiotensin system inhibitors. SMD: standardized mean difference. eGFR: estimated glomerular filtration rate. SGLT2i: sodium-glucose cotransporter-2 inhibitors. Sickle cell disease modifying therapies: any one or more of hydroxyurea, voxelotor, crizanlizumab, or apheresis/exchange transfusions/simple transfusions.

APPENDIX

ICD codes for encounter data and discharge data (at least 1 inpatient code or 2 outpatient codes).

Cardiovascular disease: coronary artery disease

410.x, 411.x, I20.0, I21.x, I24.x, I25.1x, I25.7x.

Cardiovascular disease: stroke

362.30, 362.31, 362.32, 362.33, 362.34, 433.x, 434.x, 435.x, 436.x, 431.x, G45.x, H34.1x, I63.x, I65.x, I61.x.

Diabetes mellitus

250.x, E10.x, E11.x, E12.x, E13.x, E14.x.

Heart failure

428.x, I50.x

Hypertension

997.91, 401.0, 401.1, 401.9, 402.00, 402.01, 402.10, 402.91, 402.90, 403.01, 403.00, 404.01, 404.03, 404.91, 404.92, 404.93, 404.11, 404.13, 404.12, 404.10, 402.11, 403.11, 403.10, I10.x, I11.x, I12.x, I13.x, I15.x.

Pulmonary hypertension

416.0, 416.8, 416.9, I27.0, I27.1, I27.2*

Sickle cell disease diagnosis codes

282.41, 282.42, 282.60, 282.61, 282.62, 282.63, 282.64, 282.68, 282.69, 289.52, 517.3, D57.00, D57.01, D57.02, D57.1, D57.20, D57.211, D57.212, D57.219, D57.40, D57.411, D57.412, D57.419, D57.80, D57.811, D57.812, D57.819.

Sickle cell trait diagnosis codes

282.41, D57.00

Renin angiotensin system inhibitors

candesartan (Atacand)

eprosartan (Teveten)

irbesartan (Avapro)

losartan (Cozaar)

olmesartan (Benicar)

telmisartan (Micardis)

valsartan (Diovan)

amlodipine and valsartan (Exforge)

irbesartan-hydrochlorothiazide (Avalide)

losartan potassium-hydrochlorothiazide (Hyzaar)

valsartan and hydrochlorothiazide (Diovan HCT)

benazepril (Lotensin, Lotensin Hct)

captopril (Capoten)

enalapril (Vasotec)

fosinopril (Monopril)

lisinopril (Prinivil, Zestril)

moexipril (Univasc)

perindopril (Aceon)

quinapril (Accupril)

ramipril (Altace)

trandolapril (Mavik)

Sodium-glucose co-transporter 2 inhibitors

empagliflozin (Jardiance)

canagliflozin (Invokana)

dapagliflozin (Farxiga)

Sickle cell disease modifying agents

hydroxyurea (Droxia, Hydrea)

crizanlizumab (Adakveo)

voxelotor (Oxbryta)
